# Alteration of type I interferon response is associated with subclinical atherosclerosis in virologically suppressed HIV‐1‐infected male patients

**DOI:** 10.1002/jmv.27028

**Published:** 2021-05-03

**Authors:** Letizia Santinelli, Gabriella De Girolamo, Cristian Borrazzo, Paolo Vassalini, Claudia Pinacchio, Eugenio Nelson Cavallari, Maura Statzu, Federica Frasca, Mirko Scordio, Camilla Bitossi, Agnese Viscido, Giancarlo Ceccarelli, Massimo Mancone, Claudio Maria Mastroianni, Guido Antonelli, Gabriella d'Ettorre, Carolina Scagnolari

**Affiliations:** ^1^ Department of Molecular Medicine, Laboratory of Virology, Affiliated to Istituto Pasteur Italia Sapienza University of Rome, Viale di Porta Tiburtina Rome Italy; ^2^ Department of Public Health and Infectious Diseases Sapienza University of Rome, Viale del Policlinico Rome Italy; ^3^ Department of Cardiovascular, Respiratory, Nephrology, Anaesthesiology and Geriatric Sciences Sapienza University of Rome, Viale del Policlinico Rome Italy; ^4^ Microbiology and Virology Unit Sapienza University Hospital “Policlinico Umberto I” Rome Italy

**Keywords:** atherosclerosis, cardiovascular disease, human immunodeficiency virus, IFN‐stimulated genes, innate immunity, interferon

## Abstract

Given human immunodeficiency virus‐1 (HIV‐1)‐infected patients have alterations in the type I interferon (IFN‐I) pathway and are also at elevated risk of atherosclerosis, we evaluated IFN‐I response and subclinical cardiovascular disease (CVD) association in HIV‐1‐infected patients. Transcript levels of IFN‐α/β and IFN‐stimulated gene 56 (ISG56) were evaluated by RT/real‐time PCR in peripheral blood mononuclear cells collected from asymptomatic HIV‐1‐positive male patients at high risk of developing CVD (*n* = 34) and healthy subjects (*n* = 21). Stenosis degree (≥ or <50%), calcium volume score, calcium Agatston score, and myocardial extracellular volume were examined by coronary computerized tomography scan. Carotid intima‐media thickness (cIMT), Framingham risk score, atherosclerotic cardiovascular disease (ASCVD) score, and risk score developed by data collection on adverse effects of anti‐HIV drugs (D:A:D) were also measured. Increased IFN‐α, IFN‐β, and ISG56 levels were observed in all HIV‐1‐infected males compared to healthy controls (*p* < .001 for all genes analyzed). HIV‐1‐infected patients with a stenosis degree ≥50% showed a higher Framingham risk score (*p* = .019), which was correlated with IFN‐β and ISG56 levels. HIV‐1‐infected males with enhanced IFN‐I levels and stenosis displayed a higher ASCVD calculated risk (*p* = .011) and D:A:D score (*p* = .004). Also, there was a trend toward higher IFN‐α and ISG56 mRNA levels in HIV‐1‐positive patients with an increased cIMT (*p* > .05). Dysregulation of IFN‐I response might participate in the pathogenesis of HIV‐1‐associated CVD.

## 1 INTRODUCTION

Since the introduction of combined antiretroviral therapy (cART), the life quality and survival of people living with human immunodeficiency virus‐1 (HIV‐1) have considerably enhanced.^1^ Nevertheless, as a result of the harmful effects of HIV‐1, exposure to prolonged cART and accelerated aging, cardiovascular disease (CVD) increasingly afflicts people living with HIV‐1.^2^, ^3^ The variety of CVD in HIV‐1 is not only restricted to enhanced atherosclerosis, but includes pulmonary hypertension, ventricular dysfunction related to myocarditis, complex cerebrovascular disease, pericardial pathology, endocarditis, and cardiac tumors.^4^, ^5^ The pathogenesis behind the cardiovascular HIV‐1‐associated complications is multifactorial, and involves distinct CVD risk parameters, but also viral and host factors associated with immunological and metabolic dysfunction^6^ observed during HIV‐1 infection. As a result of these observations, great interest has been directed toward the role of type I interferon (IFN‐I) in atherosclerosis.^7^, ^8^, ^9^, ^10^, ^11^, ^12^, ^13^

As HIV‐1‐infected patients have an enhanced activation of IFN‐I signalling^14^, ^15^, ^16^, ^17^ and are at elevated risk of developing atherosclerosis,^18^, ^19^ we hypothesized that persistent upregulation of IFN‐I and/or IFN‐related pathways might be involved in the development and progression of atherosclerosis during HIV‐1 infection. Therefore, we evaluated the gene expression level of IFN‐α, IFN‐β, and IFN‐stimulated gene 56 (ISG56),^20^ a well‐established marker of IFN‐I activation, in peripheral blood mononuclear cells (PBMCs) collected from HIV‐1‐infected male patients at high risk for developing CVD, examining whether alterations in IFN‐I/ISG56 mRNA levels might be associated with progression of atherosclerosis.

## 2 MATERIALS AND METHODS

### 2.1 Study population

Thirty‐four HIV‐1‐infected male patients successfully treated with cART, attending the Division of Infectious Diseases, at Department of Public Health and Infectious Diseases, “Sapienza” University of Rome (Italy) were enrolled from 2018 to 2019. Age‐ and gender‐matched healthy subjects with no history of previous severe CVD and metabolic syndrome were included as a control group (*n* = 21). For each HIV‐1‐infected patient, medical and family history, body mass index and smoke status, time from HIV‐1 diagnosis, length of cART treatment, and CD4 + T cell count were collected. To evaluate the risk of CVD, the following cardiovascular parameters were measured: Framingham 10 years risk score, atherosclerotic cardiovascular disease (ASCVD) 10 years score, data collection on adverse effects of anti‐HIV Drugs (D:A:D) 10 years estimated risk score, extracellular volume (ECV) and carotid artery intima‐media thickness (cIMT). Inclusion criteria were as follows: (1) men at least 18 years old, (2) being in cART with HIV‐1 RNA < 37 copies/ml, (3) CD4 + T cell counts >400 cells/mm^3^, (4) absence of metabolic syndrome. The study was approved by the institutional review board (Department of Public Health and Infectious Diseases, Sapienza, University of Rome) and the Ethics Committee (Sapienza, University of Rome), and all study participants signed written informed consent.

### 2.2 Multi‐detector computed tomography (MDTC) protocol and coronary angiography (CA)

All HIV‐1‐infected patients were tested for the severity of CVD using a low‐dose prospectively ECG‐triggered computerized tomography (CT) coronary angiography protocol, with a 64‐slice multidetector MDCT scanner (Somatom Definition Siemens medical Solution, Forchheimen), as previously described.^21^ Calcium score was quantified using the Agatston Calcium Score method^22^ from 3 mm nonoverlapping sections by using a semi‐automated software (calcium scoring CT; Siemens Medical Solutions). The luminal stenosis degree was classified as mild (Grade I: 30%–49%), moderate (grade II: 50%–69%), severe (Grade III: 70%–99%), or coronary occlusion (Grade IV: 100%). A threshold of 50% luminal narrowing in any coronary segment greater than 1.5 mm in diameter was adopted to define clinically significant coronary stenosis.^23^ All HIV‐1‐infected patients with coronary lesions ≥ grade II at MDCT analysis were considered for coronary angiography. This evaluation was performed using the standard technique with angiograms that were examined by an experienced blinded operator to the MDTC results. The American Heart Association (AHA) segmentation model^24^ was applied and the degree of stenosis was quantified (quantitative coronary angiography, QCA) (Allura Xper FD 10; Philips Medical Systems). Significant stenosis was defined as a reduction in diameter ≥50%.

### 2.3 CT image analysis

Pre‐ and post‐contrast Hounsfield units (HU) were measured on a Picture Archiving and Communication System. The regions of interest (ROIs) were drawn first on the equilibrium phase image at the myocardial septum and within the left ventricular chamber; the mean area was 3 cm^2^ (range, 1.5–5 cm^2^). Mean attenuation at the ROI was recorded in HU and Myocardial ECV fraction was calculated using the following equation:

ECV(%)=λ⋅(1−H)⋅100%,

where the contrast agent partition coefficient (*λ*) represents the ratio of the change in the blood and myocardial attenuation (ΔHU) and *H* is the haematocrit level. The change in attenuation (ΔHU) was determined with the following equation: ΔHU = HU_post_ − HU_pre_, where HU_post_ and HU_pre_ are attenuations after and before administration of iodinated contrast material, respectively.

### 2.4 Carotid intima‐media thickness measurement (c‐IMT)

c‐IMT measurement was performed using a B‐mode ultrasound recording with a 7‐ to 14‐MZ array probe (ESAOTE‐technology). c‐IMT was measured at a distance of at least 5 mm below the distal end of the common carotid artery. The mean value (expressed as mm) of 3 measurements was calculated for each HIV‐1‐positive patient and used as the final measurement of internal c‐IMT. Normal c‐IMT was defined as IMT < 0.9 mm and pathological c‐IMT was considered as IMT ≥ 0.9 mm. To avoid inter‐operator differences, all the measurements of c‐IMT were performed by a single operator.

### 2.5 Peripheral blood mononuclear cells isolation

Fresh peripheral blood samples (20 ml) were collected by venepuncture in Vacutainer tubes containing EDTA (BD Biosciences) from HIV‐1‐infected men and healthy individuals, and processed by Ficoll‐Hypaque density gradient centrifugation (Lympholyte, Cedarlane Labs) to obtain PBMCs. PBMCs were washed twice in phosphate‐buffered saline and stored at −80°C as dried pellets for RNA extraction.

### 2.6 Real‐time RT‐PCR assays for IFN‐α, IFN‐β, and ISG56 mRNA expression

Quantitative real‐time PCR for the analysis of IFN‐α, IFN‐β, and ISG56 mRNAs levels was carried out with the LightCycler480 instrument (Roche), as previously described.^14^, ^25^ Briefly, total RNA was extracted from PBMCs collected from HIV‐1‐infected patients and healthy individuals using a commercial RNA purification assay (Norgen Biotek Corporation) and reverse‐transcribed using the High‐Capacity cDNA Reverse Transcription Kit (Applied Biosystems), according to the manufacturer's protocol. All primers and probes were added to the Probes Master Mix (Roche, Basel, Switzerland) at 500 and 250 nm respectively, in a final volume of 20 μl. The β‐glucuronidase housekeeping gene was considered as an internal control. Gene expression values were calculated by the comparative *C*_t_ method. The primers and probe sequences used for IFN‐α, (Hs. PT.58.24294810.g) and IFN‐β (Hs. PT.58.39481063.g) were purchased from Integrated DNA Technologies (IDT). The primers and probe sequences used for ISG56 were the following: forward, 5′‐ TGAGAAGCTCTAGCCAACAACATGTC‐3′; reverse, 5′‐GAGCTTTATCCACAGAGCCTTTTC‐3′; probe 5′‐(6FAM) TATGTCTTTCGATATGCAGCCA‐AGTTTTACCG (TAM)‐3′.

### 2.7 **HIV‐1 RNA measurement and CD4** + **T lymphocyte count**

HIV‐1 viral load was determined by a Versant HIV‐1 RNA kPCR assay (Siemens Healthineers) which has a detection limit of 37 copies/ml. Absolute CD4 + T lymphocyte count was performed by FACScalibur flow cytometer (Becton Dickinson).

### 2.8 Statistical analysis

Patients data were expressed as median/IQR (interquartile range) (age, CD4 + T cell count at enrolment, CD4 + T cell count at Nadir, years from HIV‐1 diagnosis, years on ART, Framingham risk score, ASCVD score, D:A:D score, calcium score, calcium score Agatston, ECV, luminal stenosis) or as frequency (percentage) (class of anti‐HIV‐1 drug, smokers, opportunistic infections, diabetes, family history of CVD, previous CVD). The demographic and clinical characteristics of HIV‐1‐infected patients and healthy controls were compared using Student's *t* and *χ* ^2^ tests. Differences in the clinical characteristics (Framingham risk score, ASCVD risk score, D:A:D score, Calcium volume/cm^3^ score, Calcium score Agatston, Luminal stenosis, ECV range) between HIV‐1‐positive patients with a cIMT ≥ 0.9 mm and <0.9 mm were evaluated using the Mann–Whitney *U* test. Differences in IFN‐α, IFN‐β, and ISG56 mRNA levels between HIV‐1‐infected patients and healthy controls were analyzed using the Mann–Whitney *U* test. The same test was applied to evaluate any differences in the transcript level of IFN‐I genes between HIV‐1‐infected patients with coronary stenosis ≥50% and <50%, and between HIV‐1‐infected patients with cIMT ≥ 0.9 mm and <0.9 mm. Spearman's rho coefficient was calculated to assess the correlation between IFN‐α, IFN‐β, ISG56, and the Framingham score in HIV‐1‐infected patients. Statistical analyses were performed with SPSS v.25.0 for Windows: a *p* value less than .05 was considered statistically significant.

## 3 RESULTS

### 3.1 Patients characteristics

Overall, the study population included 34 cART‐treated HIV‐1‐infected males at high risk of developing CVD and 21 healthy subjects. The demographic and clinical features of the HIV‐1‐positive men and healthy controls are reported in Table 1. In particular, HIV‐1‐infected males, with a median age of 47.5 years (IQR, 40.2–51.5 years), had a median CD4 + T cell count of 448 cells/mm^3^ (IQR, 346–532.2 cells/mm^3^) and they had received cART for a median of 7.5 years (IQR, 5–10 years). Eleven out of 34 HIV‐1‐infected patients (32.4%) showed a luminal stenosis ≥ 50% while 67.6% (*n* = 23) had a luminal stenosis <50% (Table 1). HIV‐1‐infected patients with luminal stenosis ≥ 50% had a higher ASCVD calculated risk score, calcium volume and calcium Agatston scores than those with stenosis degree <50% (Table 1, *p* < .001 for all cardiovascular parameters). Opportunistic infections were detected in 45.5% (5 of 11) of HIV‐1‐infected patients with a stenosis level ≥50% compared to 39.1% (9 of 23) in those with a lower cardiovascular risk (Table 1, *p* = .002). The analysis of patients' smoking history showed that no one had never smoked.

**Table 1:** Demographic and clinical characteristics of HIV‐1‐positive males and healthy controls

Item^a^	Healthy male controls, *n* = 21	HIV‐1‐infected men, *n* = 34	Luminal stenosis		A versus B *p* value
			≥50%, *n* = 11 (A)	<50%, *n* = 23 (B)	
Age (years)	55 (42.5–60.5)	47.5 (40.2–51.5)	48 (42–54.2)	45 (40–48)	.082
HIV‐1 RNA (copies/ml)^b^	NA^c^	<37	<37	<37	1.000
CD4 + T at enrolment (cells/mm^3^)	NA	448 (346–532.2)	490 (326.5–655)	446 (376–487.5)	.236
CD4 + T nadir (cells/mm^3^)	NA	185.5 (108.2–292.5)	140 (53–229.2)	247 (155–391)	**.023**
Years from HIV‐1 diagnosis	NA	9.5 (5–10)	10 (5–10.2)	7(3–10)	.245
Years on cART	NA	7.5 (5–10)	8 (5–10)	6 (0–10)	.283
Anti‐HIV‐1 drug class, *n* (%)					
NNRTI	NA	15 (44.1)	3 (27.2)	12 (52.1)	.177
PI	NA	22 (64.7)	10 (90.9)	12 (52.1)	**.033**
ABC	NA	15 (44.1)	8 (72.7)	7 (30.4)	**.022**
Opportunistic infections, *n* (%)	NA	14 (41.1)	5 (45.5)	9 (39)	**.021**
Diabetes, *n* (%)	0 (0)	0 (0)	0 (0)	0 (0)	1.000
Family history of CVD, *n* (%)	3 (15)	2 (13)	2 (13)	3 (15)	.340
Previous CVD, *n* (%)	0 (0)	0 (0)	0 (0)	0 (0)	1.000
Framingham risk score (%)^d^	NA	3 (1–6)	4 (1–9)	3 (1–3)	**.019**
ASCVD risk score (%)^d^	NA	4 (2.8–5.9)	5.2 (3.8–8)	3 (2.8–3.9)	**.011**
D:A:D risk score (%)^d^	NA	12.2 (7.4–20.9)	18 (8.9–26.7)	8 (4–12)	**.004**
Calcium volume score (cm^3^)	NA	35.4 (10–146.95)	35.4 (10–74.3)	6.4 (0–13.6)	**<.001**
Calcium score Agatston (HU)	NA	49.2 (11–245.3)	49.2 (11–102.3)	7.0 (0–15)	**<.001**
ECV (%)	NA	30.5 (28.4–34.2)	32.3 (28.4–36.2)	29.4 (28.4–31.5)	.518

Abbreviations: ABC, abacavir; ASCVD, atherosclerotic cardiovascular disease; cART, combined antiretroviral therapy; CVD, cardiovascular disease; ECV, extracellular volume; HIV‐1, human immunodeficiency virus‐1; NNRTI, non‐nucleoside reverse transcriptase inhibitor; PI, protease inhibitor.

^a^ Data are expressed as median (IQR) or percentages. No statistically significant differences were recorded in demographic characteristics and family history of CVD between HIV‐1‐infected patients and heathy controls, *p* > .05 using Student's *t* and *χ* ^2^ tests.

^b^ HIV‐1 viral load was determined by versant HIV‐1 RNA kPCR assay (Siemens Healthcare Diagnostic), which has a detection limit of 37 copies/ml.

^c^ NA, not applicable.

^d^ Framingham 10 years risk score, ASCVD 10 years risk score and D:A:D 10 years risk score are expressed as median (IQR) of percentage values.

### 3.2 Luminal stenosis, CV risk, and IFN‐I response in HIV‐1‐infected patients

**Figure 1:**
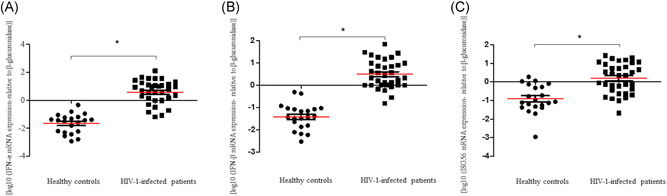
IFN‐α, IFN‐β, and ISG56 gene expression levels in HIV‐1‐infected patients and healthy controls. The mRNA levels of (A) IFN‐α, (B) IFN‐β, and (C) ISG56 were evaluated in PBMCs of 34 HIV‐1‐infected patients and 21 healthy controls; *p < .001 for all genes analyzed, determined by Mann–Whitney *U* test. IFN‐α, interferon‐α; ISG56, IFN‐stimulated gene 56; HIV‐1, human immunodeficiency virus‐1

**Figure 2:**
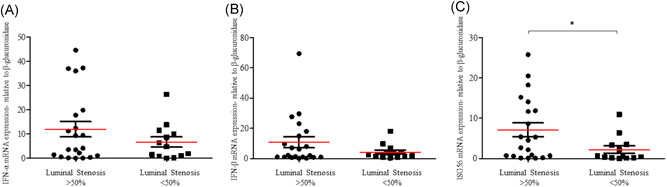
IFN‐α, IFN‐β, and ISG56 gene expression levels in HIV‐1‐infected patients with a stenosis degree ≥50% and <50%. The mRNA levels of (A) IFN‐α, (B) IFN‐β,  and (C) ISG56 were evaluated in PBMCs of HIV‐1‐infected patients with a stenosis degree ≥ 50% (*n* = 23) and <50% (*n* = 11); *p < .05, determined by Mann–Whitney *U* test. IFN‐α, interferon‐α; ISG56, IFN‐stimulated gene 56; HIV‐1, human immunodeficiency virus‐1; mRNA, messenger RNA; PBMC, peripheral blood mononuclear cell

As IFN‐I played a complex and controversial role in HIV‐1 immunopathogenesis,^16^, ^26^, ^27^ we measured the gene expression levels of IFN‐α, IFN‐β, and ISG56 in PBMCs of HIV‐1‐infected patients and healthy subjects. Results indicated that HIV‐1‐infected men have an increased amount of IFN‐α, IFN‐β, and ISG56 mRNA in PBMCs compared to healthy controls (Figure 1A–C; *p* < .001 for all genes analyzed). In particular, an average of 80‐fold increase in IFN‐α, IFN‐β, and ISG56 levels were observed in HIV‐1‐infected males (Figure 1A–C). As an elevated IFN‐I response has been related to vascular damage and CVD development,^28^ we evaluated whether the alteration in IFN‐I response might be associated with the presence of a subclinical atherosclerosis in HIV‐1‐infected men. We found that ISG56 mRNA levels were higher in HIV‐1‐infected patients with a luminal stenosis ≥50% than in those with a luminal stenosis <50% (Figure 2C, *p* = .017). A trend toward an increase in the expression of IFN‐α‐mRNA was also observed in those patients with a luminal stenosis ≥50%, however no statistically significant differences were recorded for both IFN‐α and IFN‐β (Figure 2A,B, *p* > .05). Of note, HIV‐1‐infected patients with a stenosis ≥50% showed higher Framingham risk score (Table 1, *p* = .019). Levels of this cardiological parameter were positively correlated with those of IFN‐β and ISG56 but not of IFN‐α (Table 2).

**Table 2:** Correlation between IFN‐α, IFN‐β, ISG56 and the Framingham 10 years risk score in HIV‐1‐infected men, stratified according to the grade of their luminal stenosis

	Framingham 10 years risk score			
	Luminal stenosis ≥50%		Luminal stenosis <50%	
IFN‐α mRNA	*r* = .587	*p* = .126	*r* = −.238	*p* = .313
IFN‐β mRNA	*r* = **.707**	*p* = **.048**	*r* = .110	*p* = .620
ISG56 mRNA	*r* = **.731**	*p* = **.040**	*r* = −.264	*p* = .236

*Note*: Spearman's rho coefficient was used to assess the correlation between IFN‐α, IFN‐β, ISG56, and the Framingham 10 years risk score. Significant correlations are highlighted in bold.

Abbreviations: HIV‐1, human immunodeficiency virus‐1; IFN‐α, interferon‐α; ISG56, IFN‐stimulated gene 56.

As the Framingham risk score has been associated with an underestimation of the CVD risk in HIV‐1‐infected subjects with a longer duration of cART,^29^ we compared the IFN‐I and ISG56 levels in HIV‐1‐infected patients with a luminal stenosis <50% and those with stenosis ≥50% considering D:A:D equation. Results indicated that HIV‐1‐positive patients with a stenosis level lower than <50% and decreased IFN‐α, IFN‐β, and ISG56 mRNA levels (Figure 2A–C) had a lower risk of CVD compared to those with a higher stenosis degree and IFN‐I response (Table 1, *p* = .004; Figure 2A–C).

### 3.3 cIMT level and IFN‐I expression relationship in the development of atherosclerosis

**Figure 3:**
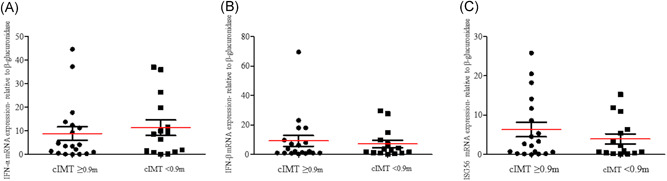
IFN‐α, IFN‐β, and ISG56 gene expression levels in HIV‐1‐infected patients with a cIMT ≥ 0.9 mm and <0.9 mm. The mRNA levels of (A) IFN‐α, (B) IFN‐β, and (C) ISG56 were evaluated in PBMCs of HIV‐1‐infected patients with a cIMT ≥ 0.9 mm (*n* = 19) and <0.9 mm (*n* = 15); *p < .05, determined by Mann–Whitney *U* test. cIMT, carotid intima‐media thickness; IFN‐α, interferon‐α; ISG56, IFN‐stimulated gene 56; HIV‐1, human immunodeficiency virus‐1; PBMC, peripheral blood mononuclear cell

cIMT is considered a surrogate measure of subclinical atherosclerosis that can predict CVD events among the general population.^30^ Thus, we examined whether the enhanced levels of IFN‐I/ISG56 and luminal stenosis found in HIV‐1‐infected men might be linked to a different cIMT degree. Nineteen (55.9%) HIV‐1‐infected males showed a cIMT ≥ 0.9 mm, a value directly associated to CVD development ^31^ (Table 3). This group of HIV‐1‐infected patients had higher calcium volume score, calcium Agatston score, and ECV compared to those with a cIMT <0.09 mm. (Table 3, *p* < .001). Furthermore, HIV‐1‐infected males with an increased cIMT showed a trend toward an upregulation of ISG56 and a deregulation of IFN‐α mRNA expression (Figure 3A,C; *p* > .05), compared to those with a cIMT <0.9 mm.

**Table 3:** Comparison of CV risk factors in HIV‐1‐infected males stratified according to the cIMT ≥ 0.9 mm or <0.9 mm

Item^a^	cIMT ≥ 0.9 mm, *n* = 19	cIMT <0.9 mm, *n* = 15	*p* value^b^
Framingham risk score (%)^c^	5 (2–9)	1 (1‐3)	<**.001**
ASCVD risk score (%)^c^	5.1 (3.6–6.4)	2.9 (2.3–3.5)	**.003**
D:A:D risk score (%)^c^	18 (10–22)	4 (3–12)	**.001**
Calcium volume score (cm^3^)	3.3 (0–35.4)	0	**<.001**
Calcium score agatston (HU)	4.9 (0–49.2)	0	**<.001**
Luminal stenosis (%)	50 (40–58)	25 (20–30)	**<.001**
ECV (%)	33.2 (28.67–35.7)	28.66 (26.8–30.4)	**.008**

Abbreviations: ASCVD, atherosclerotic cardiovascular disease; cIMT, carotid intima‐media thickness; CV, cardiovascular; ECV, extracellular volume.

^a^ Data are expressed as median (IQR) or percentage.

^b^ Differences in the clinical characteristics between HIV‐1‐positive patients with a cIMT ≥ 0.9 mm and <0.9 mm were evaluated using the Mann–Whitney *U* test.

^c^ Framingham 10 years risk score, ASCVD 10 years risk score and D:A:D 10 years risk score are expressed as median (IQR) of percentage values.

## 4 DISCUSSION

CVD represent one of the main causes of morbidity in people living with HIV‐1. Here, we evaluated whether IFN‐I response can be associated to subclinical atherosclerosis during HIV‐1 infection. As expected, IFN‐α, IFN‐β, and ISG56 transcripts were increased in PBMCs of HIV‐1‐infected patients compared to healthy controls.^14^, ^16^ To understand whether an upregulation in the IFN‐I signature might promote the development of cardiovascular damage, we examined the relationship between levels of IFN‐α/β, ISG56 and those of different cardiovascular parameters (e.g., coronary luminal stenosis, Framingham risk score, ASCVD risk score, calcium Agatston score, D:A:D risk score, cIMT and ECV). Despite the low Framingham risk score, we found a deep coronary obstruction in about 32% of HIV‐1‐infected patients, using a cut‐off value of 50% for defining significant stenosis,^32^, ^33^, ^34^ which was correlated to an increased IFN‐β and ISG56 expression in PBMCs. Interestingly, it has been reported that HIV‐1‐infected patients with a low Framingham risk score are at high risk of developing subclinical carotid atherosclerosis.^21^, ^35^ The presence of opportunistic infections have been also associated with an increased risk of developing CVD in patients with luminal stenosis ≥50%.^36^ Beyond its status as an independent risk factor, smoking increases the risk of CVD,^6^ but the analysis of smoking history showed that no‐one of HIV‐1‐infected patients had ever smoked.

More recently, IFN‐I have been shown to be a crucial modulators of atherosclerosis disease: the persistent expression of IFN‐I can promote and sustain endothelial dysfunction, which in turn contribute to atherosclerosis development in patients suffering from SLE.^37^ Specifically, IFN‐I appears to be involved in atherosclerosis development through the following mechanisms: (i) increase in macrophage recruitment^8^, ^28^; (ii) foam cell formation^9^; (iii) disruption of vascular repair^38^ and (iv) plaque progression.^37^ The absence of IFN‐I signalling can also counteract the formation of heart lesions and the recruitment of macrophages to arteries in a mouse model of atherosclerosis.^37^ Hence, the enhanced levels of IFN‐I and ISG56 observed in HIV‐1‐infected men might be caused by an excessive secretion of these cytokines by activated macrophages, given these cells are the major contributors of the atherosclerotic inflammatory response.^39^, ^40^

On the other hand, the upregulation of IFN‐I response might favour macrophage attraction toward the atherosclerotic plaque.^8^ Indeed, blocking IFN‐I signalling has been shown to decrease the macrophage accumulation at atherosclerotic plaque level.^7^, ^40^ Accordingly, it is likely that HIV‐1‐associated systemic inflammation might favor monocytes migration across the vascular endothelium; moreover, HIV‐1 infection can reduce reverse trans endothelial migration of macrophages present at the atherosclerotic level, promoting the persistence of macrophages within the plaques and increasing the risk of CVD expansion.^41^ In this complex scenario, IFN‐I response has been shown to exhibit strong pro‐ and anti‐atherogenic properties.^8^, ^9^, ^10^, ^11^, ^12^

Due to the increasing evidence of subclinical cardiovascular atherosclerosis events in young HIV‐1‐infected males compared to uninfected controls,^42^ our results also highlighted the importance of performing coronary CT scan in asymptomatic HIV‐1‐infected subjects for CVD. Although HIV‐1‐infected patients with luminal stenosis < 50% and cIMT < 0.9 mm might be considered as a control subgroup with no CVD, it could be more interesting to include a group of healthy donors with subclinical CVD to give more strength to the role played by IFN‐I response as potential predictor of increased risk of CVD in asymptomatic HIV‐1‐positive individuals; nevertheless, we are highly confident that these observations remain an interesting preliminary source of data for better understanding the relationship between persistent IFN‐I upregulation and subclinical atherosclerosis in HIV‐1‐infected patients.

In conclusion, overall, these results supported the existence of a relationship between a dysregulated expression of IFN‐α, IFN‐β, and ISG56 and the onset of atherosclerosis in long‐term‐treated HIV‐1‐infected men, suggesting that measurement of IFN‐I pathways might help for identifying those individuals who are at higher risk for CVD. Additional studies on IFN‐I signature in larger cohorts of HIV‐1‐infected patients with CVD risk factors are needed to assess any clinical correlations and to better define the importance of IFN‐I pathways in the management of atherosclerosis.

## CONFLICT OF INTERESTS

The authors declare that there are no conflict of interests.

## AUTHOR CONTRIBUTIONS

Letizia Santinelli: *investigation, data curation, formal analysis, writing—original draft*; Gabriella De Girolamo: *formal analysis, writing—original draft*; Cristian Borrazzo: *software, formal analysis, data curation*; Paolo Vassalini: *investigation, resources*; Claudia Pinacchio: *investigation, resources, data curation*; Eugenio Nelson Cavallari: *clinicical investigation, resources*; Maura Statzu: *investigation, resources, data curation*; Federica Frasca: *investigation, data curation*; Mirko Scordio: *investigation, data curation*; Camilla Bitossi: *investigation*; Agnese Viscido: *investigation*; Giancarlo Ceccarelli: *validation, visualization*; Massimo Mancone: *methodology, investigation*; Claudio Maria Mastroianni: *validation, visualization*; Guido Antonelli: *validation, visualization*; Gabriella d'Ettorre: *project administration, validation, visualization, funding acquisition*; Carolina Scagnolari: *conceptualization, validation, writing—original draft, editing and revision, visualization, supervision, project administration, funding acquisition*. All authors approved the final manuscript.

### PEER REVIEW

The peer review history for this article is available at https://publons.com/publon/10.1002/jmv.27028

## Data Availability

The data are not publicly available due to them containing information that could compromise research participant privacy/consent.
